# The Open-Air Treatment of Pulmonary Tuberculosis

**Published:** 1905-09

**Authors:** 


**The Open-Air Treatment of Pulmonary Tuberculosis.** By F. W. Burton-Fanning, M.B.Cantab. Pp. vi., 176. Illustrated. London: Cassell & Company, Ltd.

It is hardly possible to write anything new in regard to the sanatorium method of treating pulmonary consumption, but Dr. Burton-Fanning has given a succinct and very readable manual describing the main points in the modern sanatorium treatment. Not the least instructive portions are the opening chapters on the etiology and symptoms of the disease, and the author lends his support to the theory of latent tuberculosis, and considers that the bulk of mankind are infected before the conditions arise which result in the active development of tuberculosis of the lungs. In the section on "Requisites for the Treatment" much stress is laid on simpler buildings as affording all that is necessary. "Any good-sized room will do for the patient, so long as there is abundance of window-space. . . . Elaborate buildings are not necessary." Anyone desiring a reliable manual, giving a brief and unbiassed resumé of the sanatorium treatment with practical information on the different methods now in vogue, will find Burton-Fanning's book an accurate and authoritative guide.

